# Comparing the effectiveness of a breastfeeding arm sling with the normal cross-cradle hold position: A quasi-experimental study in Thailand

**DOI:** 10.18332/ejm/191253

**Published:** 2024-08-02

**Authors:** Pornsri Disorntatiwat, Sudjit Liblub, Mary Steen

**Affiliations:** Ramathibodi School of Nursing, Mahidol University, Ratchathewi, Bangkok, Thailand; Curtin School of Nursing, Faculty of Health Sciences, Curtin University, Perth, Australia

**Keywords:** breastfeeding, postpartum, mothers, newborn, interventions, normal birth

## Abstract

**INTRODUCTION:**

In Thailand, the exclusive breastfeeding rate remains low at 14% in 2019, despite the World Health Organization’s recommendation of exclusive breastfeeding for the first six months. Many mothers experience challenges such as lack of confidence, fatigue, and discomfort while breastfeeding. To address these issues, the novel arm sling innovation device was developed to provide support during breastfeeding. This study aimed to compare the effectiveness of breastfeeding using the arm sling versus the normal cross-cradle hold among first-time mothers and to evaluate their satisfaction with the breastfeeding arm sling.

**METHODS:**

A quasi-experimental crossover design was employed in the postpartum unit at Ramathibodi Hospital, Thailand, in 2022. Forty-six first-time mothers breastfed using both a breastfeeding arm sling and the normal cross-cradle hold, with a washout period in between. Breastfeeding effectiveness was measured by mothers and nurse-midwives using questionnaires, and mothers’ satisfaction with the sling was assessed. Data were evaluated using descriptive statistics and t-tests.

**RESULTS:**

The breastfeeding arm sling innovation significantly improved breastfeeding effectiveness compared to the normal cross-cradle hold, reported by both mothers (t=4.32, p<0.001) and nurse-midwives (t=8.93, p<0.001). Most mothers expressed satisfaction with the arm sling, though some design aspects, such as ease of use, require improvement.

**CONCLUSIONS:**

This study suggests that the breastfeeding arm sling can potentially improve breastfeeding experiences for first-time mothers. Further research is needed on a larger scale and in more diverse settings to confirm these findings and to improve the sling’s design for user-friendliness.

## INTRODUCTION

The benefits of breastfeeding for babies are well established, as breast milk is naturally produced, safe, clean, provides complete nutrition, and contains antibodies that help protect against many common childhood illnesses^1^. Breastfeeding increases the bonding between a mother and her infant and self-esteem for mothers from the experience. Therefore, the World Health Organization has recommended that infants should be exclusively breastfed for the first six months of life and continue breastfeeding for up to two years and beyond^2^.

Despite the many benefits of breastfeeding, rates in Thailand remain low. The World Health Assembly aims to achieve a 50% exclusive breastfeeding rate for the first 6 months by 2025^3^. However, Thailand reported an exclusive breastfeeding rate of only 14% in 2019^4^. According to the ten steps to successful breastfeeding, postpartum women should facilitate immediate initiation of breastfeeding within the first hour after birth and then encourage breastfeeding every 2–3 hours with a goal of eight sessions per 24-hour period to stimulate prolactin hormone to increase milk production^1,5^. However, there are a lot of factors that contribute to inadequate breastfeeding, such as maternal discomfort, which mothers frequently experience during the breastfeeding period^6^. Previous research has found that many mothers have a lack of belief in their ability to breastfeed, and some physical conditions and tiredness can also contribute to them having difficulties breastfeeding during the early postnatal period^7^. Additionally, other problems can contribute to the first-time mothers’ inability to breastfeed their newborns, and some have reported feeling that they have insufficient milk and difficulties latching, thus leading to shortening the duration of breastfeeding^8^. It is important to note that breastfeeding experiences can vary considerably between first-time and previously breastfeeding mothers^9^.

Positioning the baby’s body and correct latching on of the baby are essential for good attachment and successful breastfeeding^10^. A cross-cradle hold position helps to guide the baby’s head and mouth while feeding and can help to avoid poor latching, which can cause nipple redness and/or cracking^11^. It has been reported that this position is preferred by new mothers who have little experience regarding breastfeeding or for babies who have not learned how to latch on to the breast well^12^. However, repetitive, prolonged baby holding can strain the wrist, potentially causing a mother to feel overtired and experience discomfort and aching sensations in her wrist and arm during breastfeeding^13^.

Currently, there are several aids to help mothers breastfeed, such as a functional nursing bra and a specifically designed supporting pillow; these aids can help mothers continue breastfeeding. However, local new mothers have expressed that they find it difficult to hold their baby in a comfortable position continually and that their arm often aches during breastfeeding. In Thailand, midwifery practice is integrated with nursing. A nurse-midwife is a trained healthcare provider specializing in prenatal, childbirth, and postnatal care for women, which includes offering breastfeeding support and promoting maternal health^14^. The nurse-midwife’s role involves helping mothers and providing support, such as guidance and counseling on practical concerns like breastfeeding positions and how to make mothers feel comfortable and thus promote longer breastfeeding^11^.

The nurse-midwife researchers have observed the mothers’ experiences and issues during breastfeeding, such as incorrect feeding position, extreme tiredness, and wrist pain, which have been identified while working in the postnatal unit at the local Ramathibodi Hospital. Given that incorrect wrist posture during cradling while breastfeeding has been found to be associated with the development of wrist pain^13^. Therefore, there is a justification for finding new ways to help and support first-time mothers to breastfeed comfortably, reduce the risk of wrist and arm pain, and increase self-esteem for the mothers who choose to breastfeed. We aimed to assess the effectiveness of the breastfeeding arm sling innovation in supporting breastfeeding in the cross-cradle hold position compared to the normal cross-cradle hold position in first-time mothers. Additionally, we aimed to evaluate satisfaction with the breastfeeding arm sling innovation for supporting breastfeeding in the cross-cradle hold position among first-time mothers. The null hypothesis examined whether there is no difference in the mean scores for breastfeeding effectiveness between using the breastfeeding arm sling innovation and breastfeeding in a normal cross-cradle hold position (primary outcome), with mothers’ satisfaction as a secondary outcome.

## METHODS

### Study design and setting

A quasi-experimental crossover design was employed for this study, focusing on evaluating the effectiveness of the breastfeeding arm sling, a newly developed product. Given its early-stage development, we opted for a ‘proof of concept’ (POC)^15^. This approach is crucial in the initial phases of product design, helping to mitigate the risk of creating an intervention that does not meet its intended purpose^15^. Therefore, a quasi-experimental crossover design was considered an appropriate first-stage research approach to test whether the intervention can help first-time mothers breastfeed more comfortably. Additionally, due to the limited number of vaginal births in our setting, we chose a quasiexperimental crossover design over a parallel randomized controlled trial (RCT). Although a parallel RCT is generally preferred to avoid carry-over effects, it would have taken significantly longer to recruit the necessary participants^16^. The crossover design allows each mother to act as her own control, potentially achieving statistical power with fewer participants^16^. This preliminary research lays the groundwork for future studies.

This study design was chosen to compare the scores after an intervention to scores on the same measure in the same participants prior to the intervention^17^. Firstly, researchers designed and developed the breastfeeding arm sling innovation and conducted a pilot test of the innovation. Secondly, the researchers explained the study information in a step-by-step approach guided by a research protocol and specific assessment requirements to nurse-midwives (research assistants) for approximately 30–60 minutes. Nurse-midwives demonstrated how they would assess to confirm the correct procedure. Since the study was in the initial phases of designing the breastfeeding arm sling, the device was exclusively used in the postpartum unit throughout the study period.

**Figure 1:**
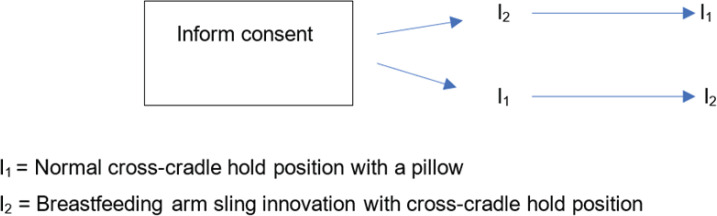
The experimental flowchart for the effectiveness of the breastfeeding arm sling in a postnatal unit of Ramathibodi Hospital, Bangkok, Thailand, 2022

The experiment involved two stages (Figure 1). In the first stage, mothers were randomly selected by drawing lots to provide 15 minutes of breastfeeding by initially using a cross-cradle hold position with the breastfeeding arm sling innovation or a normal cross-cradle hold position. As it is a clinical practice to wrap a baby (swaddle) to prevent hypothermia in the newborn in the postnatal unit at the study hospital^18,19^, the babies in this experiment were wrapped in both groups (using a cross-cradle hold position with the breastfeeding arm sling innovation or a normal cross-cradle hold position).

In the case of breastfeeding with a normal cross-cradle hold position, the nurse-midwives (research assistants) helped the mother to provide 15-minute breastfeeding. The nurse-midwives observed and measured the effectiveness of breastfeeding. In the case of breastfeeding with a cross-cradle hold position with the breastfeeding arm sling innovation, the nurse-midwives helped the mother to wear the sheath of the breastfeeding arm sling innovation and wrap the equipment around the baby. When the baby was latched on, the nurse-midwives helped the mother to adhere to the equipment for a firm hold. Following this, the mother provided 15-minute breastfeeding. The nurse-midwives observed and measured the effectiveness of breastfeeding. After breastfeeding, mothers were asked to complete the effectiveness of breastfeeding and satisfaction of using breastfeeding arm sling innovation questionnaires.

The following stage was undertaken three hours later, and 15-minute-breastfeeding was repeated (normal cross-cradle hold position or breastfeeding arm sling innovation with cross-cradle hold position). Follow-up on the effectiveness of breastfeeding and satisfaction level outcomes were assessed.

This study was conducted from January to July 2022 in the postnatal unit of Ramathibodi Hospital, Bangkok, Thailand. The hospital delivers nearly 800 babies each year, with approximately 280 vaginal births. Of these vaginal births, 200 are from first-time mothers. Two hours after giving birth, mothers were transferred to the postnatal unit and provided with care and support. It is normal practice for healthy mothers and their newborn babies to be discharged home from the postnatal unit within 48–72 hours. In this unit, assistance with breastfeeding during the postpartum period is provided by nurse-midwives. Thus, research assistants are nurse-midwives who are healthcare providers in the postnatal unit and know how to assess and observe breastfeeding behavior. Breastfeeding assistance can vary according to the nurse-midwife who provides the care. Therefore, during this study, the nurse-midwife who supported a mother to breastfeed continued to be the same person to control any breastfeeding effect. Both groups (normal cross-cradle hold position and breastfeeding arm sling innovation) received standard breastfeeding support.

### Participants and variables

A convenience sampling approach was used to recruit mothers who had vaginal births and were transferred to the postnatal unit. The criteria for inclusion were: first-time mothers with babies weighing at least 2500 g, without tongue-tie or post-delivery complications, with normal nipple size (at least 0.5 cm), and proficient in reading Thai. The research team approached these mothers within the first 72 hours post-delivery while they were in the hospital. Mothers who met these criteria were invited to participate in the study.

The study’s primary outcome is breastfeeding effectiveness, assessed by nurse-midwives and mothers. Effectiveness was defined by the baby’s position (head and body in line, close to the mother, facing the breast with nose opposite the nipple, body fully supported) and good attachment (more areola visible above the top lip, mouth wide open, lower lip turned outward, and chin touching the breast). The secondary outcome is the mother’s satisfaction with using the breastfeeding arm sling innovation, measured by a self-reported questionnaire.

The sample size was calculated based on a pilot study involving five mothers in the same setting to test the study’s feasibility (SD=1.2). According to the formula to calculate the sample size, considering an alpha error of 0.05, delta=0.5, and a test power of 80%, under one tail hypothesis, a sample size of 46 mothers was required to participate^20^.

### Intervention

The study defined the normal breastfeeding position as a cross-cradle hold with pillow support. The breastfeeding arm sling innovation was defined as an intervention that attaches to the mother’s arm to support the baby in a cross-cradle hold position with pillow support.

**Figure 2:**
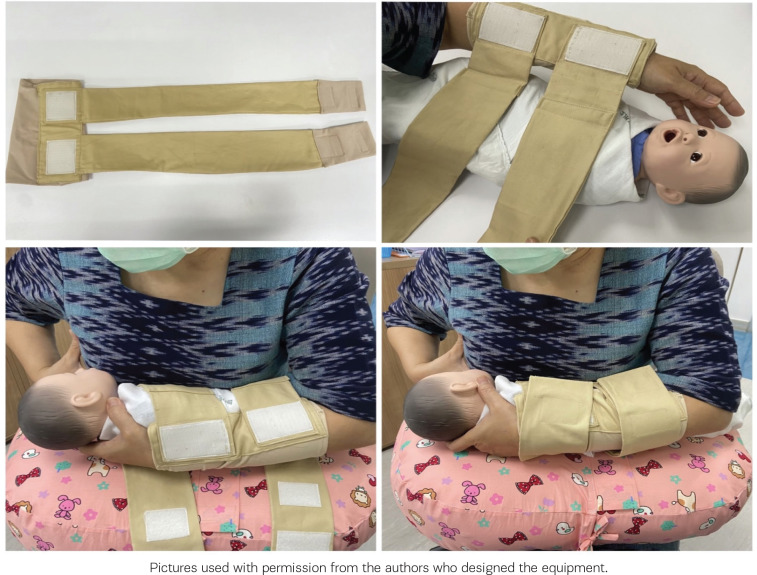
Breastfeeding arm sling innovation (above) and during use of the device (below) used in a postnatal unit of Ramathibodi Hospital, Bangkok, Thailand, 2022

The breastfeeding arm sling innovation has been designed and tailored using spandex for the sleeves, filled with elastic and attached with 6 cm leather (Figure 2). The spandex used for both arm wear is 5.0 cm apart and has two 63×7.5 cm straps. Both ends of the spandex are attached to Velcro straps in two positions to allow adjustment of the size according to the baby’s size.

In the first step of applying the support equipment, nurse-midwives helped the mother to wear the sheath of the breastfeeding arm sling innovation and wrap the equipment around the baby. When the baby was latched on and fed, nurse-midwives helped the mother adhere to the equipment for a firm hold so that the mother’s arm touched the baby’s back closely to support breastfeeding. This technique helped the mother’s arm touch the baby’s back, enabling her to hold the newborn closer. An advantage of this approach is that it prevents fatigue of the wrists and shoulders of the mothers during breastfeeding.

As this research is in the experimental stage, a helper was required to support applying the breastfeeding arm sling innovation. This initial help in using the breastfeeding arm sling innovation showed mothers how to apply and use it by themselves for further breastfeeding (Figure 2).

### Measurement

The effectiveness of the breastfeeding questionnaire was developed based on key aspects of effective breastfeeding, including the breastfeed observation’s four key points for baby positioning (head and body aligned, close to mother, facing breast with nose at nipple, whole body supported), and good attachment signs (more areola visible above upper lip, wide mouth opening, everted lower lip, chin touching breast)^21^. The participants self-reported these effective breastfeeding signs, and the nurse-midwives observed these signs. Both questionnaires included five items that utilized a ranked 5-point Likert-type scale from strongly agree to disagree, rating the effectiveness of breastfeeding outcomes strongly.

The breastfeeding arm sling innovation satisfaction questionnaire was based on the diffusion of innovation model. This model proposes that adopting an innovation is influenced by four key characteristics: compatibility, complexity, trialability, and observability^22^. The questionnaire includes 14 items ranked on a 5-point scale from lowest satisfaction to the highest level of satisfaction.

A pilot study was conducted to ensure the validity and reliability of the questionnaires. Content validity was assessed by a panel of three experts with maternal-newborn and midwifery research experience. Their recommendations led to revisions for improved question clarity. Additionally, five mothers piloted the questionnaires (separate from the main study sample). Internal consistency was evaluated using Cronbach’s alpha, which yielded a coefficient greater than 0.8, which confirmed its high reliability.

### Ethical issues

Ethics approval was obtained from the Ethics Committee on Human Subjects of the Faculty of Medicine at Ramathibodi Hospital, Mahidol University, Thailand, in November 2021 (IRB COA. MURA2021/949). The study was conducted in accordance with the ethical principles outlined in the Declaration of Helsinki^23^. Participation was entirely voluntary. All participants provided written informed consent. The researchers provided mothers with detailed written and verbal information about the research process. Confidentiality was ensured, as participants could not be identified within the data.

### Statistical analysis

All data analysis was performed using the IBM SPSS Statistics 27.0.1.0 software package^24^. The Shapiro-Wilk normality test was used to determine whether the quantitative variables’ data showed a normal distribution. Quantitative variables are presented using appropriate descriptive statistics (mean, SD). A comparison of the means of the two groups within subjects was evaluated for significance using paired t-tests. A p<0.05 was considered significant.

## RESULTS

**Figure 3:**
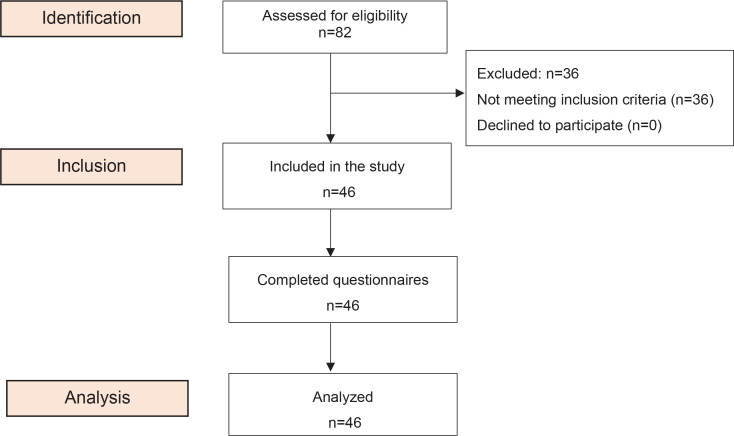
Recruitment flowchart for the evaluation of the effectiveness of the breastfeeding arm sling in a postnatal unit of Ramathibodi Hospital, Bangkok, Thailand, 2022 (N=46)

A total of eighty-two mothers who delivered vaginally were initially assessed for eligibility. Of these, 46 mothers met the inclusion criteria and consented to participate in the study. All participants completed the effectiveness of breastfeeding questionnaire for both the normal cross-cradle hold position and the breastfeeding arm sling innovation. They all completed the breastfeeding arm sling innovation satisfaction questionnaire (Figure 3).

A total of 46 postpartum women participated in the study, aged 19–40 years with a mean age of 29 years (SD=5.65). Gestational age ranged 37–41 weeks with a mean gestational age of 38 weeks (SD=1.09). Mothers who participated in the study were 18 (54.5%) on the first day postpartum, 9 (27.3 %) on the second day, and 6 (18.2%) on the third. The babies of the participants had a mean weight of 3151 g. All participants were first-time mothers with only breastfeeding experience following birth and received the hospital’s routine nursing care and newborn breastfeeding support.

The results of paired t-tests showed that the effectiveness of breastfeeding reported by mothers between using a normal cross-cradle hold position and using a cross-cradle hold position with breastfeeding arm sling innovation was statistically significantly different (t=4.32, p<0.001). The difference in total scores of using breastfeeding arm sling innovation and normal breastfeeding was statistically significant (hold the baby securely without slipping: t=5.68, p<0.001; feeling no pain in the nipples while the baby is suckling: t=4.76, p<0.001; baby can continue to suck: t=2.09, p<0.001; baby held close to mother’s body: t=5.76, p<0.001; and no pain felt in the breast during sitting to breastfeed continuously: t=4.24, p<0.001 (Table 1).

**Table 1:** Comparison of the effectiveness of breastfeeding between normal breastfeeding and using the innovation by mothers in a postnatal unit of Ramathibodi Hospital, Bangkok, Thailand, 2022 (N=46)

*Effectiveness of breastfeeding*	*Using breastfeeding arm sling innovation equipment*		*Normal breastfeeding*		*t*	*p*
	*Mean*	*SD*	*Mean*	*SD*		
Hold the baby tightly without slipping	3.24	0.67	2.20	1.02	5.68	<0.001
The feeling of no pain in the nipples while the baby is suckling	2.63	0.74	2.04	0.89	4.76	<0.001
Baby can continue to suck	3.02	0.86	2.33	0.97	2.09	<0.001
Baby held close to mother’s body	3.28	0.78	2.24	1.08	5.76	<0.001
No pain felt in the breast during sitting to breastfeed continuously	2.65	0.77	1.96	0.89	4.24	<0.001
Total	2.97	0.60	2.15	0.80	4.32	<0.001

The Shapiro-Wilk test (p<0.05).

Table 2 shows the effectiveness of breastfeeding observed by the nurse-midwives and indicates a significant difference in the effects of signs of baby attached during breastfeeding between normal breastfeeding and using breastfeeding arm sling innovation in all outcomes. More specifically, using breastfeeding arm sling innovation increased significantly as greater areola seen above the baby’s top lip (t=6.03, p<0.001); baby’s chin touches breast (t=6.26, p<0.001); baby’s head and body in line (t=7.46, p<0.001); baby’s mouth open wide (t=6.49, p<0.001); and baby’s cheeks do not dimple during breastfeed (t=7.29, p<0.001).

**Table 2:** Comparison of the effectiveness of breastfeeding between normal breastfeeding and using the innovation by nurse-midwives in a postnatal unit of Ramathibodi Hospital, Bangkok, Thailand, 2022 (N=46)

*Effectiveness of breastfeeding*	*Using breastfeeding arm sling innovation equipment*		*Normal breastfeeding*		*t*	*p*
	*Mean*	*SD*	*Mean*	*SD*		
More areola seen above baby’s top lip	2.93	0.77	2.22	0.59	6.03	<0.001
Baby’s chin touches breast	3.08	0.66	2.34	0.71	6.26	<0.001
Baby’s head and body in line	3.24	0.77	2.04	0.87	7.46	<0.001
Baby’s mouth open wide	3.24	0.82	2.37	0.77	6.49	<0.001
Baby’s cheeks do not dimple during breastfeeding	3.30	0.76	2.48	0.72	7.29	<0.001
Total	3.16	0.64	2.29	0.59	8.93	<0.001

The Shapiro-Wilk test (p<0.05).

The majority of the mothers were satisfied with the suitability of using the breastfeeding arm sling innovation design (52.9%), size and length (44.1%), and fabric used (41.2%). While half (50%) of the mothers were satisfied with the appearance of the breastfeeding arm sling innovation, 41.2% reported that they were very satisfied with the non-slippage. Meanwhile, satisfied levels of the features of easy to use (41.2%), ease of disassembly (41.2%), and ease of cleaning (38.2%) were reported by the mothers. Regarding trial-ability, 44.1% of mothers reported the breastfeeding arm sling innovation helped to reduce tension in the wrist while holding the baby. Mothers reported being satisfied with their ability to hold their baby to breastfeed (41.2%) and ease of carrying the baby (41.2%). Overall, 32.4 of mothers felt very satisfied with the use of breastfeeding arm sling innovation, and 38.2% reported feeling confident in breastfeeding (Table 3).

**Table 3:** Mothers’ satisfaction using breastfeeding arm sling innovation in a postnatal unit of Ramathibodi Hospital, Bangkok, Thailand, 2022 (N=46)

*Satisfaction reasons*	*Satisfaction levels (%)*				
	*Very Satisfied*	*Satisfied*	*Neutral*	*Unsatisfied*	*Very Unsatisfied*
**Easy-to-use features**					
Easy to use	17.6	41.2	35.3	2.9	2.9
Non slippage	41.2	41.2	17.6		
Easy to disassemble	5.9	41.2	41.2	11.8	
Easy to clean	38.2	38.2	23.5		
**Suitability of equipment**					
Design	14.7	52.9	29.4	2.9	
Size and length	26.5	44.1	26.5	2.9	
Beauty	11.8	50.0	35.3	2.9	
Fabric used	32.4	41.2	23.5	2.9	
**Trial-ability**					
The ability to hold a baby to breastfeed	35.3	41.2	17.6	5.9	
Ease of carrying a baby	35.3	41.2	17.2	5.9	
Pain in the arm while holding the baby	35.3	29.4	23.5	11.8	
Tension in the wrist while holding the baby	20.6	44.1	26.5	8.8	
**Observability**					
Satisfaction with using innovation for breastfeeding	32.4	38.2	23.5	5.9	
Feelings of confidence in breastfeeding	38.2	35.3	20.6	5.9	

## DISCUSSION

This study aimed to assess the effectiveness of the novel breastfeeding arm sling innovation in supporting breastfeeding in the cross-cradle hold position compared to the normal cross-cradle hold position and to evaluate satisfaction with the breastfeeding arm sling innovation among first-time mothers. The results indicate that breastfeeding effectiveness scores were significantly higher with the breastfeeding arm sling innovation than the normal cross-cradle hold. The breastfeeding arm sling innovation significantly improved baby positioning (head and body alignment, close to mother, facing the breast with the nose at the nipple, whole body support) and attachment signs (more areola visible above the upper lip, wide mouth opening, everted lower lip, chin touching the breast). Most mothers reported satisfaction with using the breastfeeding arm sling innovation for breastfeeding. However, further observation is needed to evaluate the device’s ease of use and improve its impact on reducing tension during breastfeeding.

Proper baby positioning and latching techniques are crucial for a good attachment and effective breastfeeding^10^. Improper latch and inappropriate infant positioning can cause nipple pain and trauma^25^. Breastfeeding difficulties commonly arise during the initial postnatal phase and usually result in discomfort^26^. Results from this study demonstrate that the breastfeeding arm sling innovation facilitates breastfeeding in first-time mothers and reduces nipple pain while the baby feeds. This improvement is potentially due to the firmer hold provided by the arm sling compared to the cross-cradle hold alone, which can be physically demanding due to the sustained use of the mother’s arm and hand^27^. These findings align with previous research by Mohammadi et al.^28^, which emphasized that the success of the breastfeeding process depends on the accuracy of the baby’s position and attachment to the mother’s breast and the baby’s ability to suck. Ensuring proper positioning and attachment during breastfeeding may lead to reduced nipple pain, increased breastfeeding duration, and fewer overall problems^29^.

Breastfeeding can be challenging for first-time mothers who have little experience of breastfeeding and newborns who have not yet learned how to latch on to the breast^12^ properly. The current study’s results show that the breastfeeding arm sling innovation helps mothers feel less pain in the arm while holding the baby and sitting to breastfeed continuously. The study’s findings are consistent with previous research demonstrating that innovations can address patient demands or help solve problems, leading to better outcomes for patients^11^. First-time mothers in this study valued the breastfeeding arm sling innovation and felt very satisfied as it helped them feel comfortable during breastfeeding. This may be due to the breastfeeding arm sling innovation fitting the mother’s arm to promote a correct position and enabling her to touch the baby’s back to hold her baby closer during breastfeeding. This aligns with existing research demonstrating the role of ergonomic support, such as breastfeeding pillows, in reducing discomfort and promoting proper breastfeeding positioning^6^. Enhanced maternal comfort reported in this study may bolster breastfeeding confidence, a critical factor influencing its sustainability early postpartum^26^. These promising initial findings serve as proof of concept for the potential of the breastfeeding arm sling to enhance maternal comfort and support breastfeeding practices. However, further large-scale research is essential to validate its efficacy and broader applicability.

The breastfeeding arm sling innovation was reported to have very satisfactory features due to its non-slipping benefit. However, the device requires improvement in usability, as mothers scored it lower on easy to use, disassembly, and wrist comfort. The current design involves strapping the baby to the mother’s arm and wrist to maintain good posture during breastfeeding, potentially limiting the mother’s flexibility in bringing the baby to the breast and affecting overall convenience. This contrasts with mothers’ natural preference to position and hold the infant for easier breastfeeding freely^30^. Effective attachment and positioning during initial feeds are crucial in preventing breastfeeding difficulties^31^. The arm sling was developed to assist first-time mothers in learning breastfeeding during their hospital stay, aiming to establish proper attachment and positioning early on, to support continued breastfeeding post-discharge. However, this innovation requires assistance to wear and disassemble, hindering its usability for self-use. Given that this is the first trial of the arm sling innovation, further studies are needed to enhance the breastfeeding support device to be more convenient for self-use and to improve its user-friendly design.

### Strengths and limitations

A strength of this study is the crossover design, which allows each mother to act as her own control, reducing selection bias by balancing out pre-existing differences between mothers when comparing their outcomes within the arm sling and control periods^16^. Furthermore, having the same nurse-midwife assess all participants minimizes confounding variables from differences in how different assessors might evaluate the outcomes^32^. However, other uncontrolled confounding factors, such as newborn feeding patterns, mothers’ breastfeeding experience, and socioeconomic factors, may have influenced the findings, limiting the inference of the research results. Further research with a more robust design, such as a randomized controlled trial (RCT) with two independent groups, is needed to strengthen the study’s ability to isolate the specific effect of the breastfeeding arm sling. Additionally, while the study had a high response rate, likely due to mothers’ enthusiasm for trying the novel breastfeeding arm sling, its generalizability may be limited by being conducted at a single hospital in Thailand. Future research in more diverse settings is necessary to determine the broader applicability of these findings.

## CONCLUSIONS

The novel breastfeeding arm sling potentially improved breastfeeding effectiveness and reduced nipple pain compared to the cross-cradle hold alone among first-time mothers. Mothers reported high satisfaction with the device, which enhanced their comfort and confidence in breastfeeding. Despite its benefits, the arm sling’s usability and ease of disassembly need improvement. Further research, including randomized controlled trials and broader studies, is required to confirm these findings and refine the device for better self-use and convenience.

## Supplementary Material



## Data Availability

All data generated or analyzed during this study are included in this article.
